# The Cat

**Published:** 1881-10

**Authors:** 


The Cat: An Introduction to the Study of Backboned Animals, especially mammals. By St. George Mivart, Ph.D., F.R.S. With 200 illustrations. New York: Charles Scribner's Sons. 1881.

The book before us contains a most complete account of the anatomy and physiology of the cat. But there is in it much more than this. The author gives briefly, but clearly, the fundamental principles of physiology in general. He also gives much of value to the student of comparative anatomy. We commend the work to persons who wish to study physiology, and to all those who are interested in natural history or comparative anatomy. It is a book that ought to interest, and cannot but instruct veterinarians.

